# Downregulation of S100 Calcium Binding Protein A9 in Esophageal Squamous Cell Carcinoma

**DOI:** 10.1155/2015/325721

**Published:** 2015-12-14

**Authors:** Harsh Pawar, Srinivas M. Srikanth, Manoj Kumar Kashyap, Gajanan Sathe, Sandip Chavan, Mukul Singal, H. C. Manju, Kariyanakatte Veeraiah Veerendra Kumar, M. Vijayakumar, Ravi Sirdeshmukh, Akhilesh Pandey, T. S. Keshava Prasad, Harsha Gowda, Rekha V. Kumar

**Affiliations:** ^1^Institute of Bioinformatics, International Technology Park, Bangalore 560066, India; ^2^Rajiv Gandhi University of Health Sciences, Bangalore 560041, India; ^3^Department of Pathology, Kidwai Memorial Institute of Oncology, Bangalore 560029, India; ^4^Department of Zoology, Savitribai Phule Pune University, Ganeshkhind, Pune, Maharashtra 411007, India; ^5^Centre of Excellence in Bioinformatics, School of Life Sciences, Pondicherry University, Pondicherry 605014, India; ^6^McKusick-Nathans Institute of Genetic Medicine, Johns Hopkins University School of Medicine, Baltimore, MD 21205, USA; ^7^Department of Biological Chemistry, Johns Hopkins University School of Medicine, Baltimore, MD 21205, USA; ^8^Moores Cancer Center, University of California, San Diego, La Jolla, CA 92093-0960, USA; ^9^Government Medical College and Hospital, Sector 32, Chandigarh 160030, India; ^10^Department of Surgical Oncology, Kidwai Memorial Institute of Oncology, Bangalore 560029, India; ^11^Department of Oncology, Johns Hopkins University School of Medicine, Baltimore, MD 21205, USA; ^12^Department of Pathology, Johns Hopkins University School of Medicine, Baltimore, MD 21205, USA

## Abstract

The development of esophageal squamous cell carcinoma (ESCC) is poorly understood and the major regulatory molecules involved in the process of tumorigenesis have not yet been identified. We had previously employed a quantitative proteomic approach to identify differentially expressed proteins in ESCC tumors. A total of 238 differentially expressed proteins were identified in that study including S100 calcium binding protein A9 (S100A9) as one of the major downregulated proteins. In the present study, we carried out immunohistochemical validation of S100A9 in a large cohort of ESCC patients to determine the expression and subcellular localization of S100A9 in tumors and adjacent normal esophageal epithelia. Downregulation of S100A9 was observed in 67% (*n* = 192) of 288 different ESCC tumors, with the most dramatic downregulation observed in the poorly differentiated tumors (99/111). Expression of S100A9 was restricted to the prickle and functional layers of normal esophageal mucosa and localized predominantly in the cytoplasm and nucleus whereas virtually no expression was observed in the tumor and stromal cells. This suggests the important role that S100A9 plays in maintaining the differentiated state of epithelium and suggests that its downregulation may be associated with increased susceptibility to tumor formation.

## 1. Introduction

Esophageal squamous cell carcinoma (ESCC) is more common in developing countries including India and China [1]. The incidence of ESCC is more common in males, with the male : female ratio being 2 : 1 [2–4]. The major risk factors associated with ESCC include alcohol consumption and tobacco usage [5–8]. Other dietary risk factors include ingestion of mycotoxins, salted food, smoked foods, and deficiency of essential micronutrients such as vitamin A and zinc [9].

*S100A9* belongs to the S100 family of genes that includes 17 members located within the epidermal differentiation complex (EDC) as a cluster on chromosome 1q21 locus [10–13]. These functionally related genes are involved in terminal epidermal differentiation [14] and loss of heterozygosity (LOH) is frequently observed at 1q21 locus [15]. S100A9 is a protein of 114 amino acids, which forms a heterodimer with another S100 protein S100A8. This 24 kDa heterodimeric complex is commonly known as calprotectin [16]. S100A9 is sometimes also referred as migration inhibitory factor related protein 14 (MRP14) or calgranulin B (CAGB).

*S100A9* has been shown to be upregulated in various cancers such as squamous cancers of the skin [17], breast ductal carcinoma [18], lung cancer [19], bladder cancer [20, 21], and prostate adenocarcinoma [22]. However,* S100A9* has been reported to be downregulated in head and neck squamous cell carcinoma (HNSCC) [23]. Various studies in the past have identified* S100A9* to be predominantly downregulated in esophageal cancer. Previous gene expression studies from other groups [24] as well as from our own group have shown* S100A9* to be downregulated in ESCC [25]. The majority of the studies have been carried out in Chinese ESCC patients and these studies provide valuable insights into the downregulation of S100 genes/proteins including S100A9 in ESCC. These studies include transcriptomic analysis [24], differential gene expression analysis [26], differential display RT-PCR [27], and immunohistochemistry [28]. Transcriptomic and proteomic studies in Indian ESCC patients have also shown downregulation of S100A9 [25, 29]. There are no reports of S100A9 IHC study on ESCC in Indian patients.

**Figure 1:**
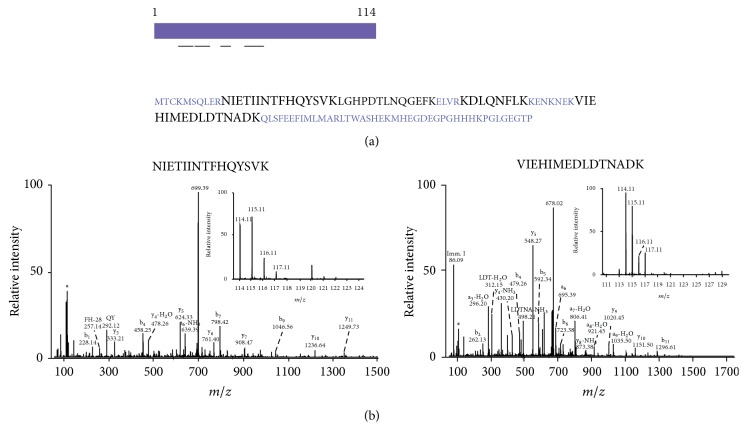
Protein sequence of S100 calcium binding protein A9 and the representative MS/MS spectra of peptides from S100 calcium binding protein A9. (a) Protein sequence of S100A9 and the peptides (in black) mapping to S100A9 protein identified in our ESCC tissue proteomics study. (b) MS/MS spectra of peptides mapping to S100A9 protein identified in ESCC tissue proteomics study. The inset shows reporter tags reflecting relatively higher expression in normal esophageal epithelia (114 and 115 reporter tags) as compared to ESCC tissue (116 and 117 reporter ions).

In total, 4 unique peptides were identified mapping to S100A9, resulting in 45% coverage of S100A9.  Figure 1 provides information regarding S100A9 protein structure and MS/MS spectra of peptides identified in the previous quantitative proteomic study [25, 29]. Hence, we selected this protein for further validation by immunohistochemical labeling of tumor and adjacent normal tissue from a larger cohort of ESCC cases.

## 2. Materials and Methods

### 2.1. Tissue Samples

Immunohistochemistry (IHC) was carried out on a large number of samples using tissue microarrays (TMAs) (*n* = 200) and tissue sections (tumor and adjacent normal epithelia) from ESCC patients of Indian origin (*n* = 100). Commercially available TMAs were obtained from following vendors: (i) US Biomax, Inc. (Rockville, MD; Catalog numbers ES1201, ES1202, and ES801) consisting of 160 ESCC cases with matched adjacent normal esophageal epithelia. The ESCC tumors varied from Grade I to Grade III and were obtained from patients in the age group of 36 to 78 years and (ii) FolioBio (Powell, OH; Catalog number ARY-HH0091) consisting of 40 ESCC cases with matched adjacent normal esophageal epithelia. The ESCC tumors varied from Grade I to Grade III and were obtained from patients in the age group of 43 to 76 years. Of the purported 200 cases on TMAs, 12 cases were found to have either missing tumor or corresponding normal core and were thus not included in the analysis. Of the 188 evaluable ESCC cases from TMAs, 29 cases were well differentiated, 113 cases were moderately differentiated, and 46 cases were poorly differentiated squamous carcinomas. Additionally, of the 100 Indian ESCC cases, 7 cases were well differentiated, 28 cases were moderately differentiated, and 65 cases were poorly differentiated squamous carcinomas.

Archived formalin-fixed paraffin embedded (FFPE) tissue blocks of ESCC samples and adjacent normal epithelium (*n* = 100) were obtained from the Department of Pathology, Kidwai Memorial Institute of Oncology (KMIO), Bangalore, India. Details of gender, age, and use of tobacco and alcohol were obtained from archived case records. Five ESCC cases were used to standardize the S100A9 antibody dilution and determine the staining pattern before large-scale validation on TMA and tissue sections. An ethical clearance was obtained from the Ethical Committee of KMIO, Bangalore.

### 2.2. Immunohistochemical Staining for Study of S100A9 Expression

A rabbit polyclonal S100A9 antibody was purchased from Santa Cruz Biotechnology (Catalog number sc-20173). According to the manufacturer, the immunogen (antigen) used to generate the S100A9 antibody was a 90-amino-acid-long, recombinant human S100A9 protein fragment mapping to the C-terminus of S100A9. The optimum dilution of the S100A9 antibody was determined on noncancerous cervical squamous epithelium obtained from the tumor-free margins of resected cervical cancer specimens, respectively. Various antibody dilutions (1 : 100, 1 : 250, 1 : 500, 1 : 1000, and 1 : 1500) were tested before deciding on 1 : 1000 as the optimal dilution based on intensity of staining as well as absence of nonspecific background staining. The staining pattern of normal esophageal squamous epithelium (*n* = 5) and ESCC tumor sections (*n* = 5) was then verified before large-scale immunostaining.

An Envision kit (Dako, Glostrup, Denmark) was used according to the manufacturer's instructions. The IHC staining procedure was carried out as previously described [30]. Briefly, the FFPE tissues were deparaffinized and subjected to antigen retrieval which was carried out by heating the tissue sections for 20 minutes at 100°C in antigen retrieval buffer (citrate buffer pH 6.0) using an electric steamer (Croma, India). This was followed by quenching of endogenous peroxidases by using the blocking solution (ready to use from Dako, Glostrup, Denmark) followed by three washes with the wash buffer. The sections were incubated with the primary antibody overnight at 4°C in a humidified chamber. After washing, the slides were incubated with horseradish peroxidase conjugated secondary antibody (Vector labs, Burlingame, CA) for 30 minutes at room temperature. The staining was developed for 5 minutes using DAB chromogen (Dako, Glostrup, Denmark), followed by counterstaining with Harris hematoxylin (Nice Chemicals, Kochi, India). Immunohistochemical staining was assessed by an experienced pathologist (RVK), who was blinded to the clinical and pathological data.

The staining was scored based on modified McCarty's *H*-scoring system, which takes into account the percentage of positive cells and the intensity of staining to provide a total score varying from 0 to 300. The “*H*-score” is a semiquantitative method of assessing the extent of immunostaining. For big tissue sections, a total of 10 fields were screened and examined at 400x magnification and in the TMA cores, all cells were counted. The staining was designated as negative (*H*-score < 49), weakly positive (1+; *H*-score of 50–99), moderately positive (2+; 100–199), or strongly positive (3+, 201–300). A comparison was made between the intensity of the staining of normal esophageal epithelium and that of carcinoma cells. Noncancerous cervical squamous epithelium sections (as suggested by the manufacturer) obtained from the tumor-free margins of resected cervical cancer specimens, respectively, were used as positive controls for S100A9. Noncancerous squamous epithelium where the diluent (phosphate buffered saline pH 7.5) was used instead of the primary antibody served as negative controls. Negative and positive controls were used with each IHC run.

The statistical significance of the differential staining observed was determined using a Chi-square test and Fischer's exact test. Results for S100A9 expression were considered statistically significant only if the *p* value was <0.05. The statistical analysis was carried out using R version R-2.13 (R Foundation for Statistical Computing, Vienna, Austria).

## 3. Results and Discussion

### 3.1. Clinical Details

In this study, we have investigated 288 ESCC cases represented by TMAs (*n* = 188) and whole tissue sections from Indian ESCC patients (*n* = 100). The mean age of all patients was 56.5 years while median age was 57 years (range 32–80 years). Of these, 200 were males and 88 were females. Further clinical details were available only for the 100 Indian patients, from patient records. Of these, 91% were from the low socioeconomic group. The use of alcohol and tobacco was noted in 26% and 45%, respectively, in men (67/100), with no record of extent of usage.

### 3.2. Expression of S100A9 in ESCC

**Figure 2:**
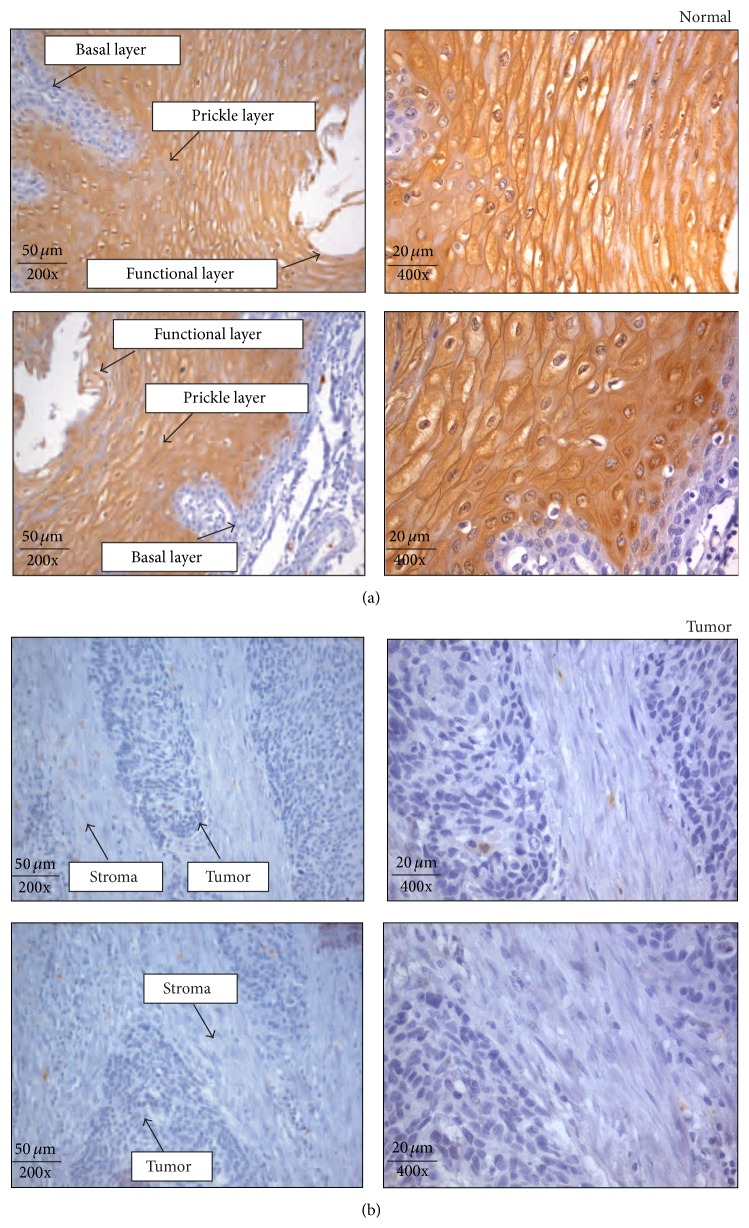
Expression of S100 calcium binding protein A9 in normal esophageal squamous epithelium and ESCC. (a) Expression of S100A9 in representative normal esophageal squamous epithelium where the expression of S100A9 (brown color) was limited to the differentiated prickle and functional layers, with no expression in the regenerative basal layer. Immunostaining pattern was cytoplasmic and nuclear. (b) Expression of S100A9 in ESCC tissue sections. S100A9 expression was undetectable in the majority of tumor cells and stroma. All the images were acquired at 200x and 400x magnification. Scale bars = 50 *μ*m and 20 *μ*m.

Expression of S100A9 in normal esophageal epithelium showed restriction of immunostaining to the prickle and functional layers. Staining was predominantly localized in the cytoplasm and nucleus of epithelial cells in these layers. The basal layer (BL) did not show expression of S100A9. Expression of S100A9 was observed in 95% (237/288) of normal esophageal epithelium (Figure 2(a)).

**Figure 3:**
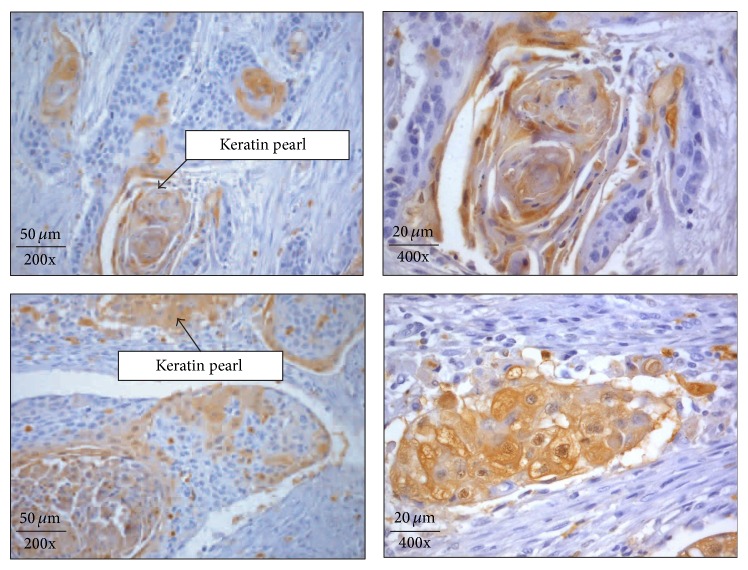
Expression of S100 calcium binding protein A9 in Grade I ESCC. S100A9 expression was observed in the foci of keratinization (keratin pearls) in Grade I tumors with cytoplasmic and nuclear localization. The degree of S100A9 immunostaining varied with the level of keratinization in tumor cells. Stroma showed no S100A9 expression. All the images were acquired at 200x and 400x magnification. Scale bars = 50 *μ*m and 20 *μ*m.

**Figure 4:**
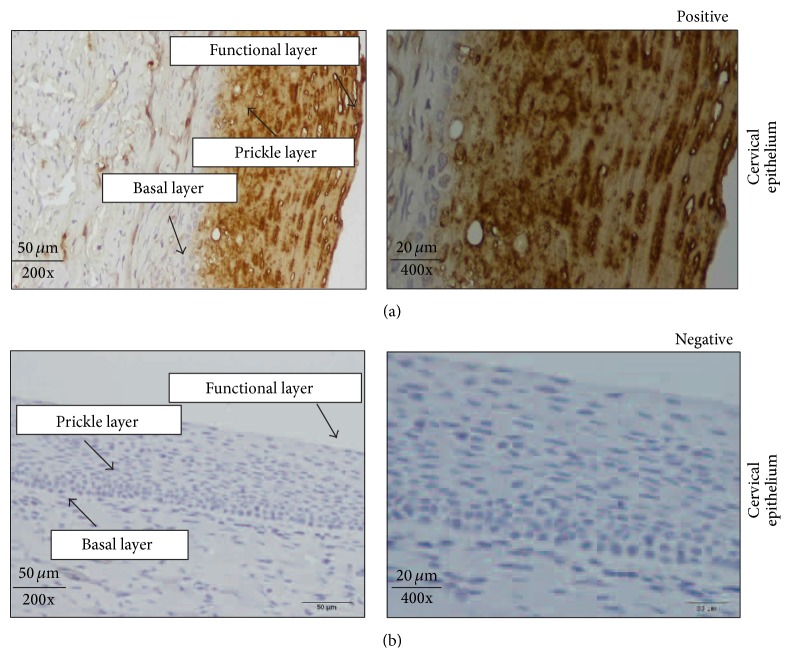
Expression of S100 calcium binding protein A9 in positive and negative controls. (a) Positive controls (normal cervical squamous epithelium) where strong to moderate expression of S100A9 was limited to the differentiated prickle and functional layers with no immunostaining of the regenerative basal layer. Expression of S100A9 was cytoplasmic and nuclear. (b) Negative controls (normal cervical squamous epithelium) without the addition of the anti-S100A9 antibody. All the images were acquired at 200x magnification and 400x. Scale bars = 50 *μ*m and 20 *μ*m.

In 89% (99/111) of Grade III ESCCs (poorly differentiated tumors), S100A9 showed no detectable or weak expression in the epithelial cells (Figure 2(b)). However, sporadic staining could be seen in 11% (12/111) in the regions that were better differentiated, depending on the amount of keratinization. Likewise, keratinized foci in the well differentiated tumors, Grades I (36/36) and II (48/141), also showed immunostaining (Figure 3). The intensity of staining varied with the level of keratinization and presence of keratin pearls. The majority of Grades I and II tumors had 30%–50% of keratinized cells within the tumor. These keratinized tumor cells showed moderate to strong expression of S100A9. Additionally, S100A9 immunostaining was not observed in the regenerative basal layer in Grade I tumors. The positive controls (normal cervical squamous epithelium) showed moderate to strong cytoplasmic and/or nuclear immunostaining (Figure 4(a)). The negative controls did not show immunostaining for S100A9 (Figure 4(b)).

### 3.3. Statistical Analysis

No correlation was seen between expression of S100A9 in tumors in relation to age and gender. There was statistically significant (*p* < 0.05) difference in the expression of S100A9 between tumors and normal tissues within a 95% confidence interval. The IHC staining pattern in tumors and normal tissues is summarized in Table 1 and the differential staining pattern of S100A9 in various histopathological Grades of ESCC is summarized in Table 2. The IHC scores for S100A9 in all ESCC cases are provided in Supplementary Table 1 (see Supplementary Material available online at http://dx.doi.org/10.1155/2015/325721).

### 3.4. Public Availability and Accessibility of IHC Data

**Figure 5:**
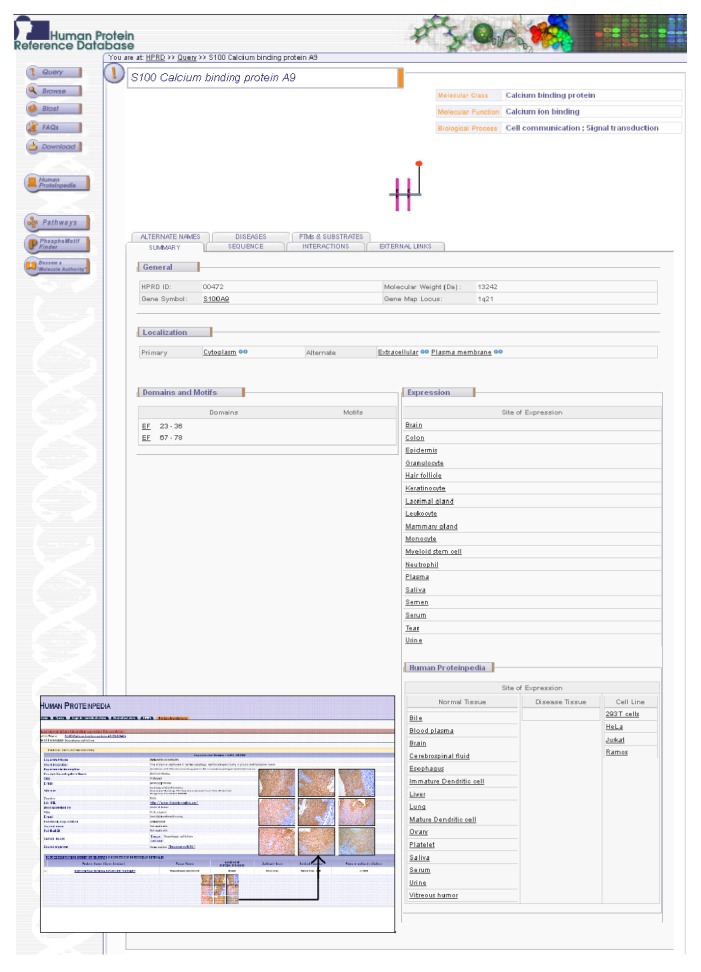
A snapshot of S100 calcium binding protein A9 annotation in Human Protein Reference Database (HPRD) and Human Proteinpedia. The molecule page of S100A9 in HPRD is shown. Also shown in the figure is the immunohistochemical labeling data for S100A9 from Human Proteinpedia, a public portal that provides data for a given protein from different experimental platforms. The figure shows the immunohistochemical staining of S100A9 in selected normal esophageal epithelial tissues.

To make our observations publicly available and accessible to other researchers, we have submitted our data on the IHC expression of S100A9 in normal esophageal epithelia (http://www.humanproteinpedia.org/Experimental_details?exp_id=TE-143441) and ESCC (http://www.humanproteinpedia.org/Experimental_details?can_id=105420) to Human Proteinpedia. The data pertaining to the peptides identified for S100A9 was also submitted to Human Proteinpedia (HUPA; http://www.humanproteinpedia.org/) [31].  Figure 5 shows a screenshot of Human Protein Reference Database (HPRD; http://www.hprd.org/) [32] page for S100A9 protein and also of the HUPA resource displaying S100A9 expression in ESCC and normal esophageal epithelia.

Normal epithelial growth and differentiation frequently involve activation and repression of genes including calcium binding S100 genes [33]. However, disruption of this process leads to dedifferentiation of epithelial cells resulting in carcinogenesis. It is becoming clear that S100A9 is involved in calcium mediated signaling pathways and may also be involved in binding to keratins [34, 35], cell cycle control [36], inflammation [37], cellular differentiation [38, 39], stress response [40], and promoting apoptosis* via* zinc sequestration [41, 42]. S100A9 has been shown to be a transcriptional target of TP53 mediated regulation and it can induce apoptosis in a TP53 dependent manner as promoter region of* S100A9* gene has been shown to have a TP53 response element. In addition, S100A9 knockdown resulted in impaired apoptosis mediated by TP53 [43]. Thus, it appears that S100A9 can regulate cell cycle progression and act as a tumor suppressor in esophageal squamous epithelium. We had previously shown the subcellular localization of another calcium binding protein cornulin (*CRNN*) to be downregulated in ESCC [30]; the role of cornulin was not known in esophageal epithelium. In a recent study by Chen et al. it was shown that cornulin is a potential tumor suppressor and it regulates cell cycle progression at G1/S checkpoint by upregulating P21^WAF1/CIP1^ and Rb. Thus it exerts its tumor suppressor effect by inhibiting G1 to S phase transition in cell cycle [44].

The esophageal squamous epithelium is constantly exposed to irritants/toxic compounds as well as pathogens such as herpes simplex virus (HSV) [45]. Stress induced by various environmental factors like zinc deficiency, alcohol consumption, nitrosamines, or pathogens may result in transient overexpression of S100A9 in epithelia as a protective inflammatory response [46]. This initial event results in increased activity of metabolic enzymes such as cyclooxygenases, which can result in metabolic activation of carcinogens. This results in mutation of* TP53*, which regulates S100A9 expression; hence S100A9 is frequently downregulated in ESCC. Apoptosis mediated cell death in disease is a protective mechanism for removing mutated cells. However, zinc deficiency may lead to disruption of this process thus resulting in ESCC tumor formation [24]. Thus S100A9 seems to play a crucial role in cell cycle progression and cellular differentiation as well as apoptosis in squamous epithelium.

In the current study, we performed immunohistochemical analysis to determine the expression of S100A9 in ESCC tumors (different Grades) and matched adjacent normal epithelia. The expression of S100A9 was found to be downregulated in the majority of poorly differentiated ESCCs (Grade III tumors). However, expression of S100A9 was restricted only to keratinized foci or keratin pearls in Grade I/II tumors with abundant cytoplasmic keratins. In case of normal esophageal epithelium, the strong cytoplasmic and nuclear expression of S100A9 was observed in the majority of esophageal epithelia and it was localized to the differentiated layers (prickle and functional). The regenerative basal layer showed no expression of S100A9. These evidences reveal that S100A9 expression is restricted to the differentiated layers and its expression is lost during the tumor development.

## 4. Conclusion

In this study, we have examined the downregulation of S100A9 at the protein level in the largest cohort of ESCC cases to date using IHC, which correlates with our previous findings from global transcriptomic and proteomic analyses. Downregulation of S100A9 in ESCC and HNSCC is in contrast to that observed in several other tumors where this protein is found to be upregulated. The present study confirms the downregulation of S100A9 in a large cohort of ESCC patients. Interestingly, S100A9 expression correlated with the histological Grade of tumor with a more dramatic downregulation observed in poorly differentiated tumors. This points to an important role that S100A9 plays in maintaining the differentiated state of epithelium and suggests that its downregulation may be associated with increased susceptibility to tumor formation. However, additional studies will be required to fully elucidate its role in the pathogenesis of ESCC.

## Supplementary Material

Supplementary Table 1 provides information regarding immunohistochemical labeling of S100A9. A large number of tissue samples using tissue microarrays (TMAs) (*n*= 200) and tissue sections (tumor and adjacent normal esophageal epithelia) from ESCC patients of Indian origin (*n*= 100) were stained for the S100A9 molecule. The table provides valuable information regarding the patient age, sex, pathology, tumor grade and TNM classification for the ESCC tumors is provided. In addition, the immunohistochemical staining information for each tissue section along with the intensity of the staining and the histological information of the tissue is provided in detail.

## Figures and Tables

**Table 1:** Summary of IHC labeling for S100 calcium binding protein A9 (S100A9) in normal and ESCC tissues.

	Parameters	Normal	ESCC
(1)	Positive	273	96
(a)	Strong (*H*-score 200–300)	84	4
(b)	Moderate (*H*-score 100–199)	112	16
(c)	Weak (*H*-score 50–99)	77	76
(2)	Negative (*H*-score 0–49)	15	192
(3)	*p* value	<0.001	

*N* = 288.

**Table 2:** Correlation between histopathological characteristics and S100A9 immunohistochemical staining in ESCC.

	Cases	No expression	Expression	*p* value
Total	288	192	96	<0.01
Age				
≥56 years	140	89	51	—
<56 years	148	105	43	
Gender				
Males	200	139	61	—
Females	88	54	34	
Pathological grade				
Well differentiated	36	—	36	<0.01
Moderately differentiated	141	93	48	
Poorly differentiated	111	99	12	

## Abbreviations

EDC: Epidermal differentiation complex
ESCC: Esophageal squamous cell carcinoma
iTRAQ: Isobaric tags for relative and absolute quantitation
LOH: Loss of heterozygosity
S100A9: S100 calcium binding protein A9.
