# Supplementary material for: Downregulation of S100 Calcium Binding Protein A9 in Esophageal Squamous Cell Carcinoma
Source: ScientificWorldJournal. 2015 Dec 14;2015:325721. doi: 10.1155/2015/325721 (PMC4691646; doi:10.1155/2015/325721)
Supplement: Supplementary file 1 — Supplementary Table 1 provides information regarding immunohistochemical labeling of S100A9. A large number of tissue samples using tissue microarrays (TMAs) (n= 200) and tissue sections (tumor and adjacent normal esophageal epithelia) from ESCC patients of Indian origin (n= 100) were stained for the S100A9 molecule. The table provides valuable information regarding the patient age, sex, pathology, tumor grade and TNM classification for the ESCC tumors is provided. In addition, the immunohistochemical staining information for each tissue section along with the intensity of the staining and the histological information of the tissue is provided in detail. [file 325721.f1.pdf]

Pawar et. al., 2013. Downregulation of calcium binding protein S100A9 in Esophageal Squamous Cell Carcinoma.
Supplementary Table 1. Summary of IHC labeling for S100A9.

Catalog	Position	Sex	Age	Pathology	Grade	TNM	Score
FolioBio	A1	M	69	Squamous cell carcinoma	I	T3N0M0	Few well differentiated keratinized tumor cell 1.5+
FolioBio	A2	M	69	Cancer adjacent normal esophageal tissue (chronic inflammation of mucosa)	-	-	1+
FolioBio	A3	M	56	Squamous cell carcinoma	I	T3N0M0	Few well differentiated keratinized tumor cell 1.5+
FolioBio	A4	M	56	Cancer adjacent normal esophageal tissue	-	-	2+
FolioBio	A5	M	62	Squamous cell carcinoma	II	T3N0M0	Dysplasia 2+
FolioBio	A6	M	62	Cancer adjacent normal esophageal tissue	-	-	1.5+
FolioBio	A7	M	58	Squamous cell carcinoma	I	T3N0M0	Few well differentiated keratinized tumor cell 1.5+
FolioBio	A8	M	58	Cancer adjacent normal esophageal tissue (chronic inflammation of mucosa)	-	-	0.5+
FolioBio	A9	M	46	Squamous cell carcinoma	I	T3N0M0	Keratinizing carcinoma 1.5+
FolioBio	A10	M	46	Cancer adjacent normal esophageal tissue	-	-	0.5+
FolioBio	B1	M	43	Squamous cell carcinoma	I	T2N0M0	Few well differentiated keratinized tumor cell 1.5+
FolioBio	B2	M	43	Cancer adjacent normal esophageal tissue	-	-	1+
FolioBio	B3	F	61	Squamous cell carcinoma	I	T3N0M0	Few well differentiated keratinized tumor cell 1+
FolioBio	B4	F	61	Cancer adjacent normal esophageal tissue	-	-	2+

Pawar et. al., 2013. Downregulation of calcium binding protein S100A9 in Esophageal Squamous Cell Carcinoma. Supplementary Table 1. Summary of IHC labeling for S100A9.

Catalog	Position	Sex	Age	Pathology	Grade	TNM	Score
FolioBio	B5	M	62	Squamous cell carcinoma	II	T2N0M0	-
FolioBio	B6	M	62	Cancer adjacent normal esophageal tissue	-	-	1.5+
FolioBio	B7	M	50	Squamous cell carcinoma	I	T2N1M0	Well differentiated keratinized tumor cell 1.5+
FolioBio	B8	M	50	Cancer adjacent normal esophageal tissue	-	-	2+
FolioBio	B9	M	68	Squamous cell carcinoma	II	T2N0M0	Few well differentiated keratinized tumor cell 1.5+
FolioBio	B10	M	68	Cancer adjacent normal esophageal tissue	-	-	2+
FolioBio	C1	M	65	Squamous cell carcinoma	I	T3N0M0	Well differentiated keratinized tumor cell 1.5+
FolioBio	C2	M	65	Cancer adjacent normal esophageal tissue	-	-	1+
FolioBio	C3	M	50	Squamous cell carcinoma	II	T2N0M0	Few well differentiated keratinized tumor cell 2+
FolioBio	C4	M	50	Cancer adjacent normal esophageal tissue	-	-	1.5+
FolioBio	C5	M	60	Squamous cell carcinoma	II	T1N0M0	-
FolioBio	C6	M	60	Cancer adjacent normal esophageal tissue	-	-	2+
FolioBio	C7	F	49	Squamous cell carcinoma	II	T3N0M0	Keratinizing carcinoma 2+
FolioBio	C8	F	49	Cancer adjacent normal esophageal tissue	-	-	1.5+
FolioBio	C9	F	59	Squamous cell carcinoma	II	T3N1M0	-
FolioBio	C10	F	59	Cancer adjacent normal esophageal tissue	-	-	1+

Pawar et. al., 2013. Downregulation of calcium binding protein S100A9 in Esophageal Squamous Cell Carcinoma. Supplementary Table 1. Summary of IHC labeling for S100A9.

Catalog	Position	Sex	Age	Pathology	Grade	TNM	Score
FolioBio	D1	M	43	Squamous cell carcinoma	II	T3N0M0	Few well differentiated keratinized tumor cell 1+
FolioBio	D2	M	43	Cancer adjacent normal esophageal tissue (chronic inflammation of mucosa)	-	-	1.5+
FolioBio	D3	M	62	Squamous cell carcinoma	II	T2N2M0	-
FolioBio	D4	M	62	Cancer adjacent normal esophageal tissue (sparse mucosa)	-	-	1.5+
FolioBio	D5	M	62	Squamous cell carcinoma	III	T2N0M0	-
FolioBio	D6	M	62	Cancer adjacent normal esophageal tissue	-	-	1.5+
FolioBio	D7	F	60	Squamous cell carcinoma	II	T3N1M0	Few well differentiated keratinized tumor cell 1+
FolioBio	D8	F	60	Cancer adjacent normal esophageal tissue (chronic inflammation of mucosa)	-	-	2+
FolioBio	D9	F	54	Squamous cell carcinoma	II	T1N0M0	-
FolioBio	D10	F	54	Cancer adjacent normal esophageal tissue	-	-	2+
FolioBio	E1	M	48	Squamous cell carcinoma	II	T3N0M0	Well differentiated keratinized tumor cell 2+
FolioBio	E2	M	48	Cancer adjacent normal esophageal tissue	-	-	1.5+
FolioBio	E3	M	57	Squamous cell carcinoma	III	T2N0M0	-
FolioBio	E4	M	57	Cancer adjacent normal esophageal tissue	-	-	2+
FolioBio	E5	M	58	Squamous cell carcinoma	II	T3N0M0	-

Pawar et. al., 2013. Downregulation of calcium binding protein S100A9 in Esophageal Squamous Cell Carcinoma. Supplementary Table 1. Summary of IHC labeling for S100A9.

Catalog	Position	Sex	Age	Pathology	Grade	TNM	Score
FolioBio	E6	M	58	Cancer adjacent normal esophageal tissue	-	-	1.5+
FolioBio	E7	F	53	Squamous cell carcinoma	III	T2N0M0	-
FolioBio	E8	F	53	Cancer adjacent normal esophageal tissue (chronic inflammation of mucosa)	-	-	1.5+
FolioBio	E9	F	56	Squamous cell carcinoma	II	T3N0M0	Few well differentiated keratinized tumor cell 1.5+
FolioBio	E10	F	56	Cancer adjacent normal esophageal tissue	-	-	2+
FolioBio	F1	M	76	Squamous cell carcinoma	II	T3N0M0	Few well differentiated keratinized tumor cell 1+
FolioBio	F2	M	76	Cancer adjacent normal esophageal tissue (chronic inflammation of mucosa)	-	-	2+
FolioBio	F3	M	60	Squamous cell carcinoma (sparse esophagus tissue)	I	T2N0M0	Well differentiated keratinized tumor cell 1.5+
FolioBio	F4	M	60	Cancer adjacent normal esophageal tissue	-	-	1.5+
FolioBio	F5	M	53	Squamous cell carcinoma	II	T3N0M0	Few well differentiated keratinized tumor cell 1+
FolioBio	F6	M	53	Cancer adjacent normal esophageal tissue	-	-	2+
**FolioBio**	**F7**	**F**	**49**	**Carcinoma in situ**	**-**	**T2N0M0**	**Dysplastic 1+**
**FolioBio**	**F8**	**F**	**49**	**Cancer adjacent normal esophageal tissue (chronic inflammation of mucosa)**	**-**	**-**	**1.5+**
FolioBio	F9	F	62	Squamous cell carcinoma	II	T3N0M0	Few well differentiated keratinized tumor cell 1.5+

Pawar et. al., 2013. Downregulation of calcium binding protein S100A9 in Esophageal Squamous Cell Carcinoma. Supplementary Table 1. Summary of IHC labeling for S100A9.							
**Catalog**	**Position**	**Sex**	**Age**	**Pathology**	**Grade**	**TNM**	**Score**
FolioBio	F10	F	62	Cancer adjacent normal esophageal tissue	-	-	2.5+
FolioBio	G1	F	48	Squamous cell carcinoma	II	T3N0M0	Few well differentiated keratinized tumor cell 2+
FolioBio	G2	F	48	Cancer adjacent normal esophageal tissue (smooth muscle and mucous gland tissue)	-	-	Very little epithelium
FolioBio	G3	M	57	Squamous cell carcinoma	III	T3N0M0	-
FolioBio	G4	M	57	Cancer adjacent normal esophageal tissue (fibrous tissue, blood vessel and smooth muscle tissue)	-	-	Very little epithelium
FolioBio	G5	M	45	Squamous cell carcinoma	III	T2N0M0	-
FolioBio	G6	M	45	Cancer adjacent normal esophageal tissue (smooth muscle tissue)	-	-	Very little epithelium
FolioBio	G7	F	55	Squamous cell carcinoma	III	T1N0M0	-
FolioBio	G8	F	55	Cancer adjacent normal esophageal tissue	-	-	1+
FolioBio	G9	F	53	Squamous cell carcinoma	III	T3N1M0	-
FolioBio	G10	F	53	Cancer adjacent normal esophageal tissue	-	-	2+
FolioBio	H1	M	68	Squamous cell carcinoma	III	T3N0M0	-
FolioBio	H2	M	68	Cancer adjacent normal esophageal tissue (chronic inflammation of mucosa)	-	-	1.5+
FolioBio	H3	M	64	Squamous cell carcinoma	II	T3N0M0	-

Pawar et. al., 2013. Downregulation of calcium binding protein S100A9 in Esophageal Squamous Cell Carcinoma.
Supplementary Table 1. Summary of IHC labeling for S100A9.

Catalog	Position	Sex	Age	Pathology	Grade	TNM	Score
FolioBio	H4	M	64	Cancer adjacent normal esophageal tissue	-	-	1+
FolioBio	H5	M	55	Squamous cell carcinoma	III	T2N0M0	-
FolioBio	H6	M	55	Cancer adjacent normal esophageal tissue	-	-	1+
FolioBio	H7	F	63	Squamous cell carcinoma	III	T3N0M0	-
FolioBio	H8	F	63	Cancer adjacent normal esophageal tissue (chronic inflammation of mucosa)	-	-	2+
FolioBio	H9	F	54	Squamous cell carcinoma	III	T3N0M0	-
FolioBio	H10	F	54	Cancer adjacent normal esophageal tissue	-	-	1.5+
U.S. Biomax	A1	M	57	Squamous cell carcinoma	II	N.A	Few well differentiated keratinized tumor cell 1.5+
U.S. Biomax	A2	M	57	Cancer adjacent tissue	-	N.A	2+
U.S. Biomax	A3	F	49	Squamous cell carcinoma	II	N.A	-
U.S. Biomax	A4	F	49	Cancer adjacent tissue (chronic inflammation)	-	N.A	1.5+
U.S. Biomax	A5	F	50	Squamous cell carcinoma (sparse)	III	N.A	-
U.S. Biomax	A6	F	50	Cancer adjacent tissue	-	N.A	2+
U.S. Biomax	A7	M	57	Squamous cell carcinoma	I	N.A	Few well differentiated keratinized tumor cell 1.5+
U.S. Biomax	A8	M	57	Cancer adjacent tissue (hyperplasia of squamous epithelium)	-	N.A	1+
U.S. Biomax	A9	M	58	Squamous cell carcinoma	II	N.A	-
U.S. Biomax	A10	M	58	Cancer adjacent tissue	-	N.A	1+
U.S. Biomax	A11	M	60	Squamous cell carcinoma	II	N.A	-
U.S. Biomax	A12	M	60	Cancer adjacent tissue (chronic inflammation)	-	N.A	1.5+

Pawar et. al., 2013. Downregulation of calcium binding protein S100A9 in Esophageal Squamous Cell Carcinoma. Supplementary Table 1. Summary of IHC labeling for S100A9.

Catalog	Position	Sex	Age	Pathology	Grade	TNM	Score
U.S. Biomax	B1	M	48	Squamous cell carcinoma	I	N.A	Few well differentiated keratinized tumor cell 2+
U.S. Biomax	B2	M	48	Cancer adjacent tissue (chronic inflammation)	-	N.A	1.5+
U.S. Biomax	B3	M	44	Squamous cell carcinoma	II	N.A	Few well differentiated keratinized tumor cell 1.5+
U.S. Biomax	B4	M	44	Cancer adjacent tissue	-	N.A	2+
U.S. Biomax	B5	M	49	Squamous cell carcinoma	II	N.A	-
U.S. Biomax	B6	M	49	Squamous cell carcinoma in situ	-	N.A	2+
U.S. Biomax	B7	M	52	Squamous cell carcinoma (sparse)	III	N.A	-
U.S. Biomax	B8	M	52	Cancer adjacent tissue (chronic inflammation)	-	N.A	1+
U.S. Biomax	B9	F	72	Squamous cell carcinoma	II	N.A	-
U.S. Biomax	B10	F	72	Cancer adjacent tissue	-	N.A	1+
U.S. Biomax	B11	F	65	Squamous cell carcinoma	I	N.A	Few well differentiated keratinized tumor cell 1+
U.S. Biomax	B12	F	65	Cancer adjacent tissue (chronic inflammation)	-	N.A	1+
U.S. Biomax	C1	M	70	Squamous cell carcinoma	III	N.A	-
U.S. Biomax	C2	M	70	Cancer adjacent tissue (chronic inflammation of smooth muscle)	-	N.A	Very little epithelium
U.S. Biomax	C3	M	72	Squamous cell carcinoma	II	N.A	-
U.S. Biomax	C4	M	72	Cancer adjacent tissue (hyperplasia of squamous epithelium)	-	N.A	2+
U.S. Biomax	C5	F	63	Squamous cell carcinoma	III	N.A	-
U.S. Biomax	C6	F	63	Cancer adjacent tissue (smooth muscle)	-	N.A	Very little epithelium
**U.S. Biomax**	**C7**	**F**	**50**	**Cancer adjacent tissue (moderate atypical hyperplasia of epithelium)**	**-**	**N.A**	**Few well differentiated keratinized tumor cell 1.5+**
**U.S. Biomax**	**C8**	**F**	**50**	**Cancer adjacent tissue (chronic inflammation with severe atypical hyperplasia of epithelium)**	**-**	**N.A**	**1+**

Pawar et. al., 2013. Downregulation of calcium binding protein S100A9 in Esophageal Squamous Cell Carcinoma. Supplementary Table 1. Summary of IHC labeling for S100A9.

Catalog	Position	Sex	Age	Pathology	Grade	TNM	Score
U.S. Biomax	C9	F	53	Squamous cell carcinoma	II	N.A	Few well differentiated keratinized tumor cell 1+
U.S. Biomax	C10	F	53	Cancer adjacent tissue	-	N.A	1+
U.S. Biomax	C11	M	53	Squamous cell carcinoma	I	N.A	Few well differentiated keratinized tumor cell 2+
U.S. Biomax	C12	M	53	Cancer adjacent tissue	-	N.A	1.5+
U.S. Biomax	D1	M	51	Squamous cell carcinoma	II	N.A	-
U.S. Biomax	D2	M	51	Cancer adjacent tissue (chronic inflammation of smooth muscle)	-	N.A	Very little epithelium
**U.S. Biomax**	**D3**	**M**	**62**	**Cancer adjacent tissue (chronic inflammation with mild atypical hyperplasia of epithelium)**	**-**	**N.A**	**-**
**U.S. Biomax**	**D4**	**M**	**62**	**Cancer adjacent tissue (chronic inflammation with hyperplasia of epithelium)**	**-**	**N.A**	**2+**
U.S. Biomax	D5	F	49	Squamous cell carcinoma	III	N.A	-
U.S. Biomax	D6	F	49	Cancer adjacent tissue	-	N.A	2+
U.S. Biomax	D7	M	55	Squamous cell carcinoma	II	N.A	-
U.S. Biomax	D8	M	55	Cancer adjacent tissue (chronic inflammation with hyperplasia of epithelium)	-	N.A	2.5+
U.S. Biomax	D9	M	50	Squamous cell carcinoma	I	N.A	Few well differentiated keratinized tumor cell 1+
U.S. Biomax	D10	M	50	Cancer adjacent tissue (chronic inflammation with hyperplasia of epithelium)	-	N.A	2+
U.S. Biomax	D11	M	43	Squamous cell carcinoma	II	N.A	-
U.S. Biomax	D12	M	43	Cancer adjacent tissue	-	N.A	2+
U.S. Biomax	E1	M	57	Squamous cell carcinoma	II	N.A	-
U.S. Biomax	E2	M	57	Cancer adjacent tissue	-	N.A	2+
**U.S. Biomax**	**E3**	**M**	**59**	**Squamous cell carcinoma**	**I**	**N.A**	**-**
**U.S. Biomax**	**E4**	**M**	**59**	**Squamous cell carcinoma**	**I**	**N.A**	**-**

Pawar et. al., 2013. Downregulation of calcium binding protein S100A9 in Esophageal Squamous Cell Carcinoma.
Supplementary Table 1. Summary of IHC labeling for S100A9.

Catalog	Position	Sex	Age	Pathology	Grade	TNM	Score
U.S. Biomax	E5	M	72	Squamous cell carcinoma	III	N.A	-
U.S. Biomax	E6	M	72	Cancer adjacent tissue	-	N.A	1.5+
U.S. Biomax	E7	M	70	Squamous cell carcinoma	III	N.A	-
U.S. Biomax	E8	M	70	Cancer adjacent tissue	-	N.A	2+
U.S. Biomax	E9	M	63	Squamous cell carcinoma (sparse)	III	N.A	-
U.S. Biomax	E10	M	63	Cancer adjacent tissue	-	N.A	2+
U.S. Biomax	E11	F	50	Squamous cell carcinoma	II	N.A	Few well differentiated keratinized tumor cell 1+
U.S. Biomax	E12	F	50	Cancer adjacent tissue (smooth muscle)	-	N.A	Very little epithelium
U.S. Biomax	F1	M	68	Squamous cell carcinoma	III	N.A	-
U.S. Biomax	F2	M	68	Cancer adjacent tissue (chronic inflammation with atypical hyperplasia of epithelium and canceration)	-	N.A	2+
U.S. Biomax	F3	F	60	Squamous cell carcinoma	III	N.A	-
U.S. Biomax	F4	F	60	Cancer adjacent tissue (chronic inflammation)	-	N.A	2+
U.S. Biomax	F5	M	65	Squamous cell carcinoma	III	N.A	-
U.S. Biomax	F6	M	65	Squamous cell carcinoma in situ	-	N.A	Dysplasia 2.5+
U.S. Biomax	F7	F	50	Squamous cell carcinoma	II	N.A	-
U.S. Biomax	F8	F	50	Cancer adjacent tissue	-	N.A	2
U.S. Biomax	F9	M	49	Early infiltrating squamous cell carcinoma	II	N.A	Few well differentiated keratinized tumor cell 1+
U.S. Biomax	F10	M	49	Cancer adjacent tissue	-	N.A	2.5+
U.S. Biomax	F11	M	58	Squamous cell carcinoma	II	N.A	-
U.S. Biomax	F12	M	58	Cancer adjacent tissue	-	N.A	2.5+
U.S. Biomax	G1	M	78	Squamous cell carcinoma	III	N.A	-
U.S. Biomax	G2	M	78	Cancer adjacent tissue (chronic inflammation)	-	N.A	2.5+
**U.S. Biomax**	**G3**	**M**	**56**	**Squamous cell carcinoma**	**III**	**N.A**	**-**
**U.S. Biomax**	**G4**	**M**	**56**	**Squamous cell carcinoma**	**III**	**N.A**	**-**
**U.S. Biomax**	**G5**	**M**	**55**	**Squamous cell carcinoma**	**II**	**N.A**	**-**

Pawar et. al., 2013. Downregulation of calcium binding protein S100A9 in Esophageal Squamous Cell Carcinoma.
Supplementary Table 1. Summary of IHC labeling for S100A9.

Catalog	Position	Sex	Age	Pathology	Grade	TNM	Score
**U.S. Biomax**	**G6**	**M**	**55**	**Squamous cell carcinoma in situ**	**-**	**N.A**	**1.5+**
U.S. Biomax	G7	M	60	Squamous cell carcinoma	III	N.A	-
U.S. Biomax	G8	M	60	Cancer adjacent tissue	-	N.A	2+
U.S. Biomax	G9	M	61	Squamous cell carcinoma	III	N.A	-
U.S. Biomax	G10	M	61	Cancer adjacent tissue (chronic inflammation)	-	N.A	2.5+
U.S. Biomax	G11	M	46	Squamous cell carcinoma	II	N.A	-
U.S. Biomax	G12	M	46	Cancer adjacent tissue (focal mild atypical hyperplasia of squamous epithelium)	-	N.A	2.5+
**U.S. Biomax**	**H1**	**M**	**57**	**Squamous cell carcinoma**	**III**	**N.A**	**-**
**U.S. Biomax**	**H2**	**M**	**57**	**Squamous cell carcinoma**	**III**	**N.A**	**-**
**U.S. Biomax**	**H3**	**M**	**57**	**Squamous cell carcinoma**	**II**	**N.A**	**-**
**U.S. Biomax**	**H4**	**M**	**57**	**Squamous cell carcinoma**	**III**	**N.A**	**-**
U.S. Biomax	H5	M	57	Squamous cell carcinoma	II	N.A	-
U.S. Biomax	H6	M	57	Cancer adjacent tissue (chronic inflammation with hyperplasia of epithelium)	-	N.A	2+
U.S. Biomax	H7	M	61	Squamous cell carcinoma	III	N.A	-
U.S. Biomax	H8	M	61	Cancer adjacent tissue	-	N.A	2+
U.S. Biomax	H9	M	48	Squamous cell carcinoma	III	N.A	-
U.S. Biomax	H10	M	48	Cancer adjacent tissue (moderate atypical hyperplasia of squamous epithelium)	-	N.A	3+
U.S. Biomax	H11	M	59	Squamous cell carcinoma	II	N.A	-
U.S. Biomax	H12	M	59	Cancer adjacent tissue (chronic inflammation with moderate atypical hyperplasia of epithelium)	-	N.A	1.5+
U.S. Biomax	I1	M	49	Squamous cell carcinoma	III	N.A	-
U.S. Biomax	I2	M	49	Cancer adjacent tissue (gland and chronic inflammation of smooth muscle)	-	N.A	2+

Pawar et. al., 2013. Downregulation of calcium binding protein S100A9 in Esophageal Squamous Cell Carcinoma. Supplementary Table 1. Summary of IHC labeling for S100A9.

Catalog	Position	Sex	Age	Pathology	Grade	TNM	Score
**U.S. Biomax**	**I3**	**M**	**62**	**Squamous cell carcinoma**	**III**	**N.A**	**-**
**U.S. Biomax**	**I4**	**M**	**62**	**Squamous cell carcinoma**	**III**	**N.A**	**-**
U.S. Biomax	I5	M	72	Squamous cell carcinoma	III	N.A	-
U.S. Biomax	I6	M	72	Cancer adjacent tissue (chronic inflammation)	-	N.A	2+
U.S. Biomax	I7	M	74	Squamous cell carcinoma	III	N.A	-
U.S. Biomax	I8	M	74	Cancer adjacent tissue (chronic inflammation)	-	N.A	1+
U.S. Biomax	I9	M	58	Squamous cell carcinoma	II	N.A	-
U.S. Biomax	I10	M	58	Cancer adjacent tissue	-	N.A	2.5+
U.S. Biomax	I11	M	73	Squamous cell carcinoma	III	N.A	-
U.S. Biomax	I12	M	73	Cancer adjacent tissue (chronic inflammation with hyperplasia of epithelium)	-	N.A	2.5+
**U.S. Biomax**	**J1**	**F**	**57**	**Carcinosarcoma**	**-**	**N.A**	**-**
U.S. Biomax	J2	F	57	Cancer adjacent tissue (smooth muscle)	-	N.A	Very little epithelium
**U.S. Biomax**	**J3**	**M**	**50**	**Malignant tumor from epithelium**	**-**	**N.A**	**-**
**U.S. Biomax**	**J4**	**M**	**50**	**Cancer adjacent tissue**	**-**	**N.A**	**2+**
U.S. Biomax	J5	M	71	Squamous cell carcinoma	III	N.A	-
U.S. Biomax	J6	M	71	Cancer adjacent tissue (chronic inflammation)	-	N.A	2+
U.S. Biomax	J7	M	49	Squamous cell carcinoma	III	N.A	-
U.S. Biomax	J8	M	49	Cancer adjacent tissue	-	N.A	1.5+
U.S. Biomax	J9	M	65	Squamous cell carcinoma	III	N.A	-
U.S. Biomax	J10	M	65	Cancer adjacent tissue	-	N.A	2+
U.S. Biomax	J11	M	56	Squamous cell carcinoma	III	N.A	-
U.S. Biomax	J12	M	56	Cancer adjacent tissue (hyperplasia of squamous epithelium)	-	N.A	1.5+
U.S. Biomax	A1	M	56	Squamous cell carcinoma	IIa	T2N0M0	Few well differentiated keratinized tumor cell 2+

Pawar et. al., 2013. Downregulation of calcium binding protein S100A9 in Esophageal Squamous Cell Carcinoma. Supplementary Table 1. Summary of IHC labeling for S100A9.

Catalog	Position	Sex	Age	Pathology	Grade	TNM	Score
U.S. Biomax	A2	M	56	Cancer adjacent tissue (fibrous tissue and smooth muscle)	-	-	Very little epithelium
U.S. Biomax	A3	M	60	Squamous cell carcinoma	IIa	T3N0M0	-
U.S. Biomax	A4	M	60	Cancer adjacent tissue (chronic inflammation with hyperplasia of epithelium)	-	-	2+
U.S. Biomax	A5	M	66	Squamous cell carcinoma	IIa	T3N0M0	-
U.S. Biomax	A6	M	66	Cancer adjacent tissue (reflux esophagitis)	-	-	2+
U.S. Biomax	A7	F	72	Squamous cell carcinoma (necrotic tissue)	IIa	T2N0M0	-
U.S. Biomax	A8	F	72	Cancer adjacent tissue (mild chronic inflammation)	-	-	3+
U.S. Biomax	A9	F	55	Squamous cell carcinoma	IIa	T3N0M0	Few well differentiated keratinized tumor cell 1+
U.S. Biomax	A10	F	55	Cancer adjacent tissue (sparse)	-	-	3+
U.S. Biomax	A11	M	61	Squamous cell carcinoma	IIa	T3N0M0	-
U.S. Biomax	A12	M	61	Cancer adjacent tissue (chronic inflammation)	-	-	2+
U.S. Biomax	B1	M	36	Squamous cell carcinoma	IIa	T3N0M0	-
U.S. Biomax	B2	M	36	Cancer adjacent tissue (chronic inflammation with hyperplasia of epithelium)	-	-	2+
U.S. Biomax	B3	F	42	Squamous cell carcinoma	IIa	T2N0M0	1+
U.S. Biomax	B4	F	42	Cancer adjacent tissue (chronic inflammation with hyperplasia of epithelium)	-	-	2+

Pawar et. al., 2013. Downregulation of calcium binding protein S100A9 in Esophageal Squamous Cell Carcinoma. Supplementary Table 1. Summary of IHC labeling for S100A9.

Catalog	Position	Sex	Age	Pathology	Grade	TNM	Score
U.S. Biomax	B5	M	54	Squamous cell carcinoma	IIa	T2N0M0	-
U.S. Biomax	B6	M	54	Cancer adjacent tissue (reflux esophagitis)	-	-	2+
U.S. Biomax	B7	F	55	Squamous cell carcinoma	Ia	T1N0M0	Few well differentiated keratinized tumor cell 1+
U.S. Biomax	B8	F	55	Cancer adjacent tissue (reflux esophagitis)	-	-	1.5+
U.S. Biomax	B9	M	57	Squamous cell carcinoma (chronic inflammation of fibrous tissue and smooth muscle)	IIa	T2N0M0	-
U.S. Biomax	B10	M	57	Cancer adjacent tissue (chronic inflammation with hyperplasia of epithelium)	-	-	1+
U.S. Biomax	B11	F	57	Squamous cell carcinoma	IIa	T3N0M0	-
U.S. Biomax	B12	F	57	Cancer adjacent tissue (fibrous tissue and blood vessel)	-	-	Very little epithelium
U.S. Biomax	C1	M	64	Squamous cell carcinoma	IIa	T3N0M0	-
U.S. Biomax	C2	M	64	Cancer adjacent tissue	-	-	3+
U.S. Biomax	C3	M	48	Squamous cell carcinoma	IIIa	T3N1M0	-
U.S. Biomax	C4	M	48	Cancer adjacent tissue (chronic inflammation)	-	-	2+
U.S. Biomax	C5	M	52	Squamous cell carcinoma	IIa	T3N0M0	-
U.S. Biomax	C6	M	52	Cancer adjacent tissue (fibrous tissue and blood vessel)	-	-	1+
U.S. Biomax	C7	F	54	Squamous cell carcinoma	IIa	T3N0M0	-

Pawar et. al., 2013. Downregulation of calcium binding protein S100A9 in Esophageal Squamous Cell Carcinoma. Supplementary Table 1. Summary of IHC labeling for S100A9.							
**Catalog**	**Position**	**Sex**	**Age**	**Pathology**	**Grade**	**TNM**	**Score**
U.S. Biomax	C8	F	54	Cancer adjacent tissue (reflux esophagitis)	-	-	2+
U.S. Biomax	C9	M	50	Squamous cell carcinoma	IIa	T3N0M0	-
U.S. Biomax	C10	M	50	Cancer adjacent tissue (chronic inflammation)	-	-	2+
U.S. Biomax	C11	F	62	Squamous cell carcinoma	IIa	T2N0M0	Few well differentiated keratinized tumor cell 1.5+
U.S. Biomax	C12	F	62	Cancer adjacent tissue (hyperplasia)	-	-	2+
U.S. Biomax	D1	M	41	Squamous cell carcinoma	IIa	T2N0M0	-
U.S. Biomax	D2	M	41	Cancer adjacent tissue	-	-	3+
**U.S. Biomax**	**D3**	**F**	**54**	**Squamous cell carcinoma (sparse)**	**-**	**TxN0M0**	**-**
**U.S. Biomax**	**D4**	**F**	**54**	**Cancer adjacent tissue (reflux esophagitis)**	**-**	**-**	**2.5+**
U.S. Biomax	D5	M	51	Squamous cell carcinoma	IIa	T2N0M0	Few well differentiated keratinized tumor cell 1.5+
U.S. Biomax	D6	M	51	Cancer adjacent tissue (chronic inflammation with mild atypical hyperplasia of epithelium)	-	-	2+
U.S. Biomax	D7	F	48	Squamous cell carcinoma	IIIa	T3N1M0	-
U.S. Biomax	D8	F	48	Cancer adjacent tissue (chronic inflammation)	-	-	2+
U.S. Biomax	D9	F	49	Squamous cell carcinoma	IIa	T2N0M0	-
U.S. Biomax	D10	F	49	Cancer adjacent tissue (chronic inflammation)	-	-	3+
U.S. Biomax	D11	M	45	Squamous cell carcinoma	IIa	T3N0M0	-
U.S. Biomax	D12	M	45	Cancer adjacent tissue (hyperplasia)	-	-	2+

Pawar et. al., 2013. Downregulation of calcium binding protein S100A9 in Esophageal Squamous Cell Carcinoma. Supplementary Table 1. Summary of IHC labeling for S100A9.

Catalog	Position	Sex	Age	Pathology	Grade	TNM	Score
U.S. Biomax	E1	M	57	Squamous cell carcinoma	IIa	T2N0M0	-
U.S. Biomax	E2	M	57	Cancer adjacent tissue (fibrous tissue and smooth muscle)	-	-	1+
U.S. Biomax	E3	M	58	Squamous cell carcinoma	IIa	T3N0M0	Few well differentiated keratinized tumor cell 1.5+
U.S. Biomax	E4	M	58	Cancer adjacent tissue	-	-	3+
U.S. Biomax	E5	M	76	Squamous cell carcinoma (fibrous tissue and smooth muscle)	IIa	T3N0M0	-
U.S. Biomax	E6	M	76	Cancer adjacent tissue (hyperplasia)	-	-	2.5+
U.S. Biomax	E7	M	51	Squamous cell carcinoma	IIa	T3N0M0	1.5+
U.S. Biomax	E8	M	51	Cancer adjacent tissue	-	-	3+
U.S. Biomax	E9	M	60	Squamous cell carcinoma	IIa	T3N0M0	Few well differentiated keratinized tumor cell 1.5+
U.S. Biomax	E10	M	60	Cancer adjacent tissue (fibrous tissue and smooth muscle)	-	-	1+
U.S. Biomax	E11	F	57	Squamous cell carcinoma	IIa	T2N0M0	-
U.S. Biomax	E12	F	57	Cancer adjacent tissue (chronic inflammation)	-	-	2+
U.S. Biomax	F1	M	62	Squamous cell carcinoma	IIa	T2N0M0	-
U.S. Biomax	F2	M	62	Cancer adjacent tissue (hyperplasia)	-	-	3+
U.S. Biomax	F3	F	60	Squamous cell carcinoma	IIa	T3N0M0	Few well differentiated keratinized tumor cell 1.5+
U.S. Biomax	F4	F	60	Cancer adjacent tissue	-	-	2.5+
U.S. Biomax	F5	M	62	Squamous cell carcinoma (sparse)	IIa	T3N0M0	-
U.S. Biomax	F6	M	62	Cancer adjacent tissue (reflux esophagitis)	-	-	2+

Pawar et. al., 2013. Downregulation of calcium binding protein S100A9 in Esophageal Squamous Cell Carcinoma. Supplementary Table 1. Summary of IHC labeling for S100A9.

Catalog	Position	Sex	Age	Pathology	Grade	TNM	Score
U.S. Biomax	F7	M	69	Squamous cell carcinoma	IIa	T3N0M0	-
U.S. Biomax	F8	M	69	Cancer adjacent tissue (chronic inflammation)	-	-	2+
U.S. Biomax	F9	M	48	Squamous cell carcinoma	IIa	T3N0M0	-
U.S. Biomax	F10	M	48	Cancer adjacent tissue (hyperplasia)	-	-	2+
U.S. Biomax	F11	M	66	Squamous cell carcinoma	IIa	T3N0M0	-
U.S. Biomax	F12	M	66	Cancer adjacent tissue (fibrous tissue and smooth muscle)	-	-	Very little epithelium
U.S. Biomax	G1	M	53	Squamous cell carcinoma	IIa	T3N0M0	Few well differentiated keratinized tumor cell 1.5+
U.S. Biomax	G2	M	53	Cancer adjacent tissue (chronic inflammation)	-	-	1+
U.S. Biomax	G3	M	72	Squamous cell carcinoma	IIa	T2N0M0	-
U.S. Biomax	G4	M	72	Cancer adjacent tissue (chronic inflammation)	-	-	2+
U.S. Biomax	G5	M	57	Squamous cell carcinoma	IIIa	T3N1M0	-
U.S. Biomax	G6	M	57	Cancer adjacent tissue (reflux esophagitis)	-	-	1+
U.S. Biomax	G7	M	63	Squamous cell carcinoma	IIa	T3N0M0	-
U.S. Biomax	G8	M	63	Cancer adjacent tissue	-	-	1+
U.S. Biomax	G9	F	53	Squamous cell carcinoma	IIIa	T3N1M0	-
U.S. Biomax	G10	F	53	Cancer adjacent tissue (chronic inflammation with hyperplasia of epithelium)	-	-	2+

Pawar et. al., 2013. Downregulation of calcium binding protein S100A9 in Esophageal Squamous Cell Carcinoma. Supplementary Table 1. Summary of IHC labeling for S100A9.

Catalog	Position	Sex	Age	Pathology	Grade	TNM	Score
U.S. Biomax	G11	M	54	Squamous cell carcinoma	IIa	T2N0M0	Few well differentiated keratinized tumor cell 1.5+
U.S. Biomax	G12	M	54	Cancer adjacent tissue (hyperplasia)	-	-	2+
U.S. Biomax	H1	M	58	Squamous cell carcinoma	IIa	T3N0M0	-
U.S. Biomax	H2	M	58	Cancer adjacent tissue (chronic inflammation)	-	-	2+
U.S. Biomax	H3	F	65	Squamous cell carcinoma	IIa	T3N0M0	-
U.S. Biomax	H4	F	65	Cancer adjacent tissue (chronic inflammation)	-	-	1+
U.S. Biomax	H5	M	54	Squamous cell carcinoma	IIIa	T3N1M0	-
U.S. Biomax	H6	M	54	Cancer adjacent tissue	-	-	3+
U.S. Biomax	H7	M	61	Squamous cell carcinoma	IIa	T3N0M0	-
U.S. Biomax	H8	M	61	Cancer adjacent tissue (chronic inflammation with mild atypical hyperplasia of epithelium)	-	-	2+
U.S. Biomax	H9	M	65	Squamous cell carcinoma	IIa	T3N0M0	-
U.S. Biomax	H10	M	65	Cancer adjacent tissue (chronic inflammation)	-	-	1+
U.S. Biomax	H11	F	60	Squamous cell carcinoma (smooth muscle)	Ia	T1N0M0	-
U.S. Biomax	H12	F	60	Cancer adjacent tissue (hyperplasia)	-	-	2+
U.S. Biomax	I1	F	72	Squamous cell carcinoma	IIa	T2N0M0	-
U.S. Biomax	I2	F	72	Cancer adjacent tissue (reflux esophagitis)	-	-	1+
U.S. Biomax	I3	M	62	Squamous cell carcinoma	IIa	T3N0M0	Few well differentiated keratinized tumor cell 1.5+

Pawar et. al., 2013. Downregulation of calcium binding protein S100A9 in Esophageal Squamous Cell Carcinoma.
Supplementary Table 1. Summary of IHC labeling for S100A9.

Catalog	Position	Sex	Age	Pathology	Grade	TNM	Score
U.S. Biomax	I4	M	62	Cancer adjacent tissue	-	-	2+
U.S. Biomax	I5	M	62	Squamous cell carcinoma	IIa	T3N0M0	-
U.S. Biomax	I6	M	62	Cancer adjacent tissue	-	-	3+
U.S. Biomax	I7	M	64	Squamous cell carcinoma	IIa	T3N0M0	Few well differentiated keratinized tumor cell 2+
U.S. Biomax	I8	M	64	Cancer adjacent tissue	-	-	3+
U.S. Biomax	I9	M	52	Squamous cell carcinoma	IIa	T3N0M0	-
U.S. Biomax	I10	M	52	Cancer adjacent tissue (gastric cardia)	-	-	Very little epithelium
U.S. Biomax	I11	F	51	Squamous cell carcinoma	IIa	T3N0M0	-
U.S. Biomax	I12	F	51	Cancer adjacent tissue (reflux esophagitis)	-	-	2+
U.S. Biomax	J1	F	66	Squamous cell carcinoma	IIa	T3N0M0	1+
U.S. Biomax	J2	F	66	Cancer adjacent tissue (chronic inflammation)	-	-	1+
U.S. Biomax	J3	F	41	Squamous cell carcinoma (sparse)	IIa	T2N0M0	-
U.S. Biomax	J4	F	41	Cancer adjacent tissue	-	-	2+
U.S. Biomax	J5	M	50	Squamous cell carcinoma	IIa	T3N0M0	-
U.S. Biomax	J6	M	50	Cancer adjacent tissue (chronic inflammation)	-	-	2+
U.S. Biomax	J7	M	46	Squamous cell carcinoma	IIa	T3N0M0	Few well differentiated keratinized tumor cell 2+
U.S. Biomax	J8	M	46	Cancer adjacent tissue (chronic inflammation)	-	-	2+
U.S. Biomax	J9	M	60	Squamous cell carcinoma	IIIa	T3N1M0	-

Pawar et. al., 2013. Downregulation of calcium binding protein S100A9 in Esophageal Squamous Cell Carcinoma. Supplementary Table 1. Summary of IHC labeling for S100A9.

Catalog	Position	Sex	Age	Pathology	Grade	TNM	Score
U.S. Biomax	J10	M	60	Cancer adjacent tissue	-	-	1+
U.S. Biomax	J11	M	65	Carcinosarcoma	IIa	T3N0M 0	-
U.S. Biomax	J12	M	65	Cancer adjacent tissue (chronic inflammation)	-	-	2+
U.S. Biomax	A1	F	49	Squamous cell carcinoma	I	N.A	Well differentiated keratinized tumor cell 1.5+
U.S. Biomax	A2	F	49	Matched normal Matched normal esophagus tissue	-	N.A	2.5+
U.S. Biomax	A3	M	65	Squamous cell carcinoma	I	N.A	Well differentiated keratinized tumor cell 1.5+
U.S. Biomax	A4	M	65	Matched normal Matched normal esophagus tissue	-	N.A	2+
U.S. Biomax	A5	M	43	Squamous cell carcinoma	I	N.A	Few well differentiated keratinized tumor cell 2+
U.S. Biomax	A6	M	43	Matched normal esophagus tissue of the above	-	N.A	2.5+
U.S. Biomax	A7	F	56	Squamous cell carcinoma	II	N.A	Few well differentiated keratinized tumor cell 1+
U.S. Biomax	A8	F	56	Mucosa chronic inflammation of esophagus	-	N.A	1.5+
U.S. Biomax	A9	M	45	Squamous cell carcinoma	II	N.A	-
U.S. Biomax	A10	M	45	Mucosa chronic inflammation of esophagus	-	N.A	2.5+
U.S. Biomax	B1	M	46	Squamous cell carcinoma	I	N.A	Few well differentiated keratinized tumor cell 1.5+
U.S. Biomax	B2	M	46	Matched normal esophagus tissue of the above	-	N.A	3+
U.S. Biomax	B3	F	53	Squamous cell carcinoma	III	N.A	-
U.S. Biomax	B4	F	53	Mucosa chronic inflammation of esophagus	-	N.A	2+

Pawar et. al., 2013. Downregulation of calcium binding protein S100A9 in Esophageal Squamous Cell Carcinoma. Supplementary Table 1. Summary of IHC labeling for S100A9.

Catalog	Position	Sex	Age	Pathology	Grade	TNM	Score
U.S. Biomax	B5	M	69	Squamous cell carcinoma	II	N.A	Few well differentiated keratinized tumor cell 1+
U.S. Biomax	B6	M	69	Mucosa chronic inflammation of esophagus	-	N.A	3+
U.S. Biomax	B7	M	68	Squamous cell carcinoma	II	N.A	-
U.S. Biomax	B8	M	68	Matched normal esophagus tissue of the above	-	N.A	2+
U.S. Biomax	B9	F	53	Squamous cell carcinoma	III	N.A	-
U.S. Biomax	B10	F	53	Matched normal esophagus tissue of the above	-	N.A	2+
U.S. Biomax	C1	F	61	Squamous cell carcinoma	I	N.A	Few well differentiated keratinized tumor cell 1+
U.S. Biomax	C2	F	61	Matched normal esophagus tissue of the above	-	N.A	2.5+
U.S. Biomax	C3	F	62	Squamous cell carcinoma	II	N.A	Few well differentiated keratinized tumor cell 1+
U.S. Biomax	C4	F	62	Matched normal esophagus tissue of the above	-	N.A	3+
U.S. Biomax	C5	M	62	Squamous cell carcinoma	I	N.A	Few well differentiated keratinized tumor cell 2+
U.S. Biomax	C6	M	62	Matched normal esophagus tissue of the above	-	N.A	2.5+
U.S. Biomax	C7	M	50	Squamous cell carcinoma	I	N.A	Few well differentiated keratinized tumor cell 1+
U.S. Biomax	C8	M	50	Matched normal esophagus tissue of the above	-	N.A	2.5+
U.S. Biomax	C9	F	49	Squamous cell carcinoma	II	N.A	-
U.S. Biomax	C10	F	49	Matched normal esophagus tissue of the above	-	N.A	2+
U.S. Biomax	D1	M	56	Squamous cell carcinoma	I	N.A	Few well differentiated keratinized tumor cell 1+

Pawar et. al., 2013. Downregulation of calcium binding protein S100A9 in Esophageal Squamous Cell Carcinoma.
Supplementary Table 1. Summary of IHC labeling for S100A9.

Catalog	Position	Sex	Age	Pathology	Grade	TNM	Score
U.S. Biomax	D2	M	56	Mucosa chronic inflammation of esophagus	-	N.A	2.5+
U.S. Biomax	D3	M	62	Squamous cell carcinoma	II	N.A	Few well differentiated keratinized tumor cell 2.5+
U.S. Biomax	D4	M	62	Matched normal esophagus tissue of the above	-	N.A	2.5+
U.S. Biomax	D5	M	65	Squamous cell carcinoma	II	N.A	-
U.S. Biomax	D6	M	65	Matched normal esophagus tissue of the above	-	N.A	2.5+
U.S. Biomax	D7	M	62	Squamous cell carcinoma	I	N.A	Few well differentiated keratinized tumor cell 1+
U.S. Biomax	D8	M	62	Matched normal esophagus tissue of the above	-	N.A	2.5+
U.S. Biomax	D9	F	59	Squamous cell carcinoma	II	N.A	-
U.S. Biomax	D10	F	59	Matched normal esophagus tissue of the above	-	N.A	1+
U.S. Biomax	E1	M	68	Squamous cell carcinoma	II	N.A	Few well differentiated keratinized tumor cell 1+
U.S. Biomax	E2	M	68	Matched normal esophagus tissue of the above	-	N.A	2+
U.S. Biomax	E3	M	57	Squamous cell carcinoma	II	N.A	-
U.S. Biomax	E4	M	57	Matched normal esophagus tissue of the above	-	N.A	2.5+
U.S. Biomax	E5	M	60	Squamous cell carcinoma	II	N.A	Few well differentiated keratinized tumor cell 2.5+
U.S. Biomax	E6	M	60	Matched normal esophagus tissue of the above	-	N.A	2.5+
U.S. Biomax	E7	M	58	Squamous cell carcinoma	I	N.A	Few well differentiated keratinized tumor cell 2+
U.S. Biomax	E8	M	58	Matched normal esophagus tissue of the above	-	N.A	2.5+

Pawar et. al., 2013. Downregulation of calcium binding protein S100A9 in Esophageal Squamous Cell Carcinoma. Supplementary Table 1. Summary of IHC labeling for S100A9.

Catalog	Position	Sex	Age	Pathology	Grade	TNM	Score
U.S. Biomax	E9	M	58	Squamous cell carcinoma	I	N.A	Few well differentiated keratinized tumor cell 1+
U.S. Biomax	E10	M	58	Matched normal esophagus tissue of the above	-	N.A	2.5+
U.S. Biomax	F1	F	55	Squamous cell carcinoma	III	N.A	-
U.S. Biomax	F2	F	55	Mucosa chronic inflammation of esophagus	-	N.A	2+
U.S. Biomax	F3	F	54	Squamous cell carcinoma	II	N.A	-
U.S. Biomax	F4	F	54	Matched normal esophagus tissue of the above	-	N.A	2.5+
U.S. Biomax	F5	M	50	Squamous cell carcinoma	II	N.A	Few well differentiated keratinized tumor cell 1+
U.S. Biomax	F6	M	50	Matched normal esophagus tissue of the above	-	N.A	2.5+
U.S. Biomax	F7	M	48	Squamous cell carcinoma	II	N.A	-
U.S. Biomax	F8	M	48	Matched normal esophagus tissue of the above	-	N.A	3+
U.S. Biomax	F9	M	57	Squamous cell carcinoma	II	N.A	Few well differentiated keratinized tumor cell 1+
U.S. Biomax	F10	M	57	Matched normal esophagus tissue of the above	-	N.A	2.5+
U.S. Biomax	G1	M	65	Squamous cell carcinoma	I	N.A	Few well differentiated keratinized tumor cell 1+
U.S. Biomax	G2	M	65	Matched normal esophagus tissue of the above	-	N.A	2+
U.S. Biomax	G3	M	62	Squamous cell carcinoma	II	N.A	-
U.S. Biomax	G4	M	62	Matched normal esophagus tissue of the above	-	N.A	2.5+
U.S. Biomax	G5	F	60	Squamous cell carcinoma	II	N.A	-
U.S. Biomax	G6	F	60	Matched normal esophagus tissue of the above	-	N.A	2.5+

Pawar et. al., 2013. Downregulation of calcium binding protein S100A9 in Esophageal Squamous Cell Carcinoma. Supplementary Table 1. Summary of IHC labeling for S100A9.

Catalog	Position	Sex	Age	Pathology	Grade	TNM	Score
U.S. Biomax	G7	F	48	Squamous cell carcinoma	I	N.A	Few well differentiated keratinized tumor cell 2+
U.S. Biomax	G8	F	48	Matched normal esophagus tissue of the above	-	N.A	3+
U.S. Biomax	G9	F	63	Squamous cell carcinoma	II	N.A	Few well differentiated keratinized tumor cell 1+
U.S. Biomax	G10	F	63	Matched normal esophagus tissue of the above	-	N.A	3+
U.S. Biomax	H1	M	60	Squamous cell carcinoma	III	N.A	-
U.S. Biomax	H2	M	60	Matched normal esophagus tissue of the above	-	N.A	2+
U.S. Biomax	H3	M	55	Squamous cell carcinoma	II	N.A	-
U.S. Biomax	H4	M	55	Matched normal esophagus tissue of the above	-	N.A	2.5+
U.S. Biomax	H5	M	53	Squamous cell carcinoma	II	N.A	-
U.S. Biomax	H6	M	53	Mucosa chronic inflammation of esophagus	-	N.A	3
U.S. Biomax	H7	M	64	Squamous cell carcinoma	II	N.A	-
U.S. Biomax	H8	M	64	Matched normal esophagus tissue of the above	-	N.A	1+
U.S. Biomax	H9	F	54	Squamous cell carcinoma	III	N.A	-
U.S. Biomax	H10	F	54	Matched mucosa chronic inflammation of esophagus	-	N.A	1+
Indian Patient 1	-	F	45	Cancer adjacent tissue	-	N.A	3+
Indian Patient 1	-	F	45	Squamous cell carcinoma	II	N.A	Few well differentiated keratinized tumor cell 2+
Indian Patient 2	-	F	50	Cancer adjacent tissue	-	N.A	2+
Indian Patient 2	-	F	50	Squamous cell carcinoma	III	N.A	-
Indian Patient 3	-	M	61	Cancer adjacent tissue	-	N.A	2+
Indian Patient 3	-	M	61	Squamous cell carcinoma	III	N.A	-
Indian Patient 4	-	M	48	Cancer adjacent tissue	-	N.A	Very little epithelium
Indian Patient 4	-	M	48	Squamous cell carcinoma	II	N.A	-

Pawar et. al., 2013. Downregulation of calcium binding protein S100A9 in Esophageal Squamous Cell Carcinoma. Supplementary Table 1. Summary of IHC labeling for S100A9.							
**Catalog**	**Position**	**Sex**	**Age**	**Pathology**	**Grade**	**TNM**	**Score**
Indian Patient 5	-	F	52	Cancer adjacent tissue	-	N.A	2.5+
Indian Patient 5	-	F	52	Squamous cell carcinoma	III	N.A	-
Indian Patient 6	-	F	55	Cancer adjacent tissue	-	N.A	2.5+
Indian Patient 6	-	F	55	Squamous cell carcinoma	III	N.A	-
Indian Patient 7	-	F	45	Cancer adjacent tissue	-	N.A	2.5+
Indian Patient 7	-	F	45	Squamous cell carcinoma	I	N.A	2+
Indian Patient 8	-	M	48	Cancer adjacent tissue	-	N.A	3+
Indian Patient 8	-	M	48	Squamous cell carcinoma	III	N.A	-
Indian Patient 9	-	F	43	Cancer adjacent tissue	-	N.A	3+
Indian Patient 9	-	F	43	Squamous cell carcinoma	II	N.A	-
Indian Patient 10	-	F	35	Cancer adjacent tissue	-	N.A	3+
Indian Patient 10	-	F	35	Squamous cell carcinoma	III	N.A	Few well differentiated keratinized tumor cell 1+
Indian Patient 11	-	F	55	Cancer adjacent tissue	-	N.A	3+
Indian Patient 11	-	F	55	Squamous cell carcinoma	III	N.A	-
Indian Patient 12	-	F	34	Cancer adjacent tissue	-	N.A	2+
Indian Patient 12	-	F	34	Squamous cell carcinoma	III	N.A	Few well differentiated keratinized tumor cell 1.5+
Indian Patient 13	-	M	41	Cancer adjacent tissue	-	N.A	3+
Indian Patient 13	-	M	41	Squamous cell carcinoma	I	N.A	Few well differentiated keratinized tumor cell 1+
Indian Patient 14	-	F	77	Cancer adjacent tissue	-	N.A	3+
Indian Patient 14	-	F	77	Squamous cell carcinoma	III	N.A	-
Indian Patient 15	-	F	49	Cancer adjacent tissue	-	N.A	3+
Indian Patient 15	-	F	49	Squamous cell carcinoma	III	N.A	-
Indian Patient 16	-	F	32	Cancer adjacent tissue	-	N.A	1.5+
Indian Patient 16	-	F	32	Squamous cell carcinoma	I	N.A	Few well differentiated keratinized tumor cell 1+
Indian Patient 17	-	M	60	Cancer adjacent tissue	-	N.A	2+
Indian Patient 17	-	M	60	Squamous cell carcinoma	I	N.A	Few well differentiated keratinized tumor cell 1.5+
Indian Patient 18	-	M	55	Cancer adjacent tissue	-	N.A	3+

Pawar et. al., 2013. Downregulation of calcium binding protein S100A9 in Esophageal Squamous Cell Carcinoma. Supplementary Table 1. Summary of IHC labeling for S100A9.

Catalog	Position	Sex	Age	Pathology	Grade	TNM	Score
Indian Patient 18	-	M	55	Squamous cell carcinoma	II	N.A	-
Indian Patient 19	-	F	70	Cancer adjacent tissue	-	N.A	Very little epithelium
Indian Patient 19	-	F	70	Squamous cell carcinoma	III	N.A	Few well differentiated keratinized tumor cell 1.5+
Indian Patient 20	-	M	52	Cancer adjacent tissue	-	N.A	1.5+
Indian Patient 20	-	M	52	Squamous cell carcinoma	II	N.A	-
Indian Patient 21	-	M	60	Cancer adjacent tissue	-	N.A	3+
Indian Patient 21	-	M	60	Squamous cell carcinoma	III	N.A	-
Indian Patient 22	-	F	55	Cancer adjacent tissue	-	N.A	3+
Indian Patient 22	-	F	55	Squamous cell carcinoma	III	N.A	-
Indian Patient 23	-	M	70	Cancer adjacent tissue	-	N.A	3+
Indian Patient 23	-	M	70	Squamous cell carcinoma	III	N.A	Few well differentiated keratinized tumor cell 1.5+
Indian Patient 24	-	F	57	Cancer adjacent tissue	-	N.A	2+
Indian Patient 24	-	F	57	Squamous cell carcinoma	II	N.A	-
Indian Patient 25	-	M	68	Cancer adjacent tissue	-	N.A	2.5+
Indian Patient 25	-	M	68	Squamous cell carcinoma	III	N.A	-
Indian Patient 26	-	F	65	Cancer adjacent tissue	-	N.A	3+
Indian Patient 26	-	F	65	Squamous cell carcinoma	III	N.A	Few well differentiated keratinized tumor cell 1+
Indian Patient 27	-	M	62	Cancer adjacent tissue	-	N.A	2.5+
Indian Patient 27	-	M	62	Squamous cell carcinoma	III	N.A	-
Indian Patient 28	-	M	55	Cancer adjacent tissue	-	N.A	2.5+
Indian Patient 28	-	M	55	Squamous cell carcinoma	II	N.A	-
Indian Patient 29	-	M	55	Cancer adjacent tissue	-	N.A	3+
Indian Patient 29	-	M	55	Squamous cell carcinoma	III	N.A	-
Indian Patient 30	-	M	35	Cancer adjacent tissue	-	N.A	3+
Indian Patient 30	-	M	35	Squamous cell carcinoma	III	N.A	-
Indian Patient 31	-	M	60	Cancer adjacent tissue	-	N.A	2+
Indian Patient 31	-	M	60	Squamous cell carcinoma	III	N.A	-
Indian Patient 32	-	F	55	Cancer adjacent tissue	-	N.A	2+

Pawar et. al., 2013. Downregulation of calcium binding protein S100A9 in Esophageal Squamous Cell Carcinoma. Supplementary Table 1. Summary of IHC labeling for S100A9.

Catalog	Position	Sex	Age	Pathology	Grade	TNM	Score
Indian Patient 32	-	F	55	Squamous cell carcinoma	III	N.A	Few well differentiated keratinized tumor cell 1+
Indian Patient 33	-	M	40	Cancer adjacent tissue	-	N.A	3+
Indian Patient 33	-	M	40	Squamous cell carcinoma	II	N.A	-
Indian Patient 34	-	M	55	Cancer adjacent tissue	-	N.A	1.5+
Indian Patient 34	-	M	55	Squamous cell carcinoma	III	N.A	-
Indian Patient 35	-	M	52	Cancer adjacent tissue	-	N.A	1.5+
Indian Patient 35	-	M	52	Squamous cell carcinoma	I	N.A	Few well differentiated keratinized tumor cell 2+
Indian Patient 36	-	M	58	Cancer adjacent tissue	-	N.A	1.5+
Indian Patient 36	-	M	58	Squamous cell carcinoma	II	N.A	-
Indian Patient 37	-	M	65	Cancer adjacent tissue	-	N.A	2.5+
Indian Patient 37	-	M	65	Squamous cell carcinoma	III	N.A	-
Indian Patient 38	-	M	52	Cancer adjacent tissue	-	N.A	1+
Indian Patient 38	-	M	52	Squamous cell carcinoma	III	N.A	-
Indian Patient 39	-	M	45	Cancer adjacent tissue	-	N.A	3+
Indian Patient 39	-	M	45	Squamous cell carcinoma	III	N.A	-
Indian Patient 40	-	M	60	Cancer adjacent tissue	-	N.A	3+
Indian Patient 40	-	M	60	Squamous cell carcinoma	II	N.A	Few well differentiated keratinized tumor cell 1.5+
Indian Patient 41	-	M	59	Cancer adjacent tissue	-	N.A	3+
Indian Patient 41	-	M	59	Squamous cell carcinoma	II	N.A	-
Indian Patient 42	-	M	70	Cancer adjacent tissue	-	N.A	2+
Indian Patient 42	-	M	70	Squamous cell carcinoma	III	N.A	-
Indian Patient 43	-	M	50	Cancer adjacent tissue	-	N.A	1.5+
Indian Patient 43	-	M	50	Squamous cell carcinoma	II	N.A	-
Indian Patient 44	-	M	55	Cancer adjacent tissue	-	N.A	2+
Indian Patient 44	-	M	55	Squamous cell carcinoma	II	N.A	-
Indian Patient 45	-	M	70	Cancer adjacent tissue	-	N.A	1+
Indian Patient 45	-	M	70	Squamous cell carcinoma	III	N.A	-
Indian Patient 46	-	M	36	Cancer adjacent tissue	-	N.A	2.5+
Indian Patient 46	-	M	36	Squamous cell carcinoma	II	N.A	-

Pawar et. al., 2013. Downregulation of calcium binding protein S100A9 in Esophageal Squamous Cell Carcinoma.
Supplementary Table 1. Summary of IHC labeling for S100A9.

Catalog	Position	Sex	Age	Pathology	Grade	TNM	Score
Indian Patient 47	-	M	68	Cancer adjacent tissue	-	N.A	2+
Indian Patient 47	-	M	68	Squamous cell carcinoma	III	N.A	-
Indian Patient 48	-	F	80	Cancer adjacent tissue	-	N.A	1+
Indian Patient 48	-	F	80	Squamous cell carcinoma	II	N.A	-
Indian Patient 49	-	M	60	Cancer adjacent tissue	-	N.A	2+
Indian Patient 49	-	M	60	Squamous cell carcinoma	III	N.A	-
Indian Patient 50	-	F	50	Cancer adjacent tissue	-	N.A	2.5+
Indian Patient 50	-	F	50	Squamous cell carcinoma	III	N.A	-
Indian Patient 51	-	F	65	Cancer adjacent tissue	-	N.A	1.5+
Indian Patient 51	-	F	65	Squamous cell carcinoma	III	N.A	Few well differentiated keratinized tumor cell 1.5+
Indian Patient 52	-	M	68	Cancer adjacent tissue	-	N.A	1+
Indian Patient 52	-	M	68	Squamous cell carcinoma	II	N.A	-
Indian Patient 53	-	M	42	Cancer adjacent tissue	-	N.A	1+
Indian Patient 53	-	M	42	Squamous cell carcinoma	III	N.A	-
Indian Patient 54	-	F	60	Cancer adjacent tissue	-	N.A	2+
Indian Patient 54	-	F	60	Squamous cell carcinoma	III	N.A	-
Indian Patient 55	-	M	40	Cancer adjacent tissue	-	N.A	2+
Indian Patient 55	-	M	40	Squamous cell carcinoma	III	N.A	Few well differentiated keratinized tumor cell 1+
Indian Patient 56	-	M	66	Cancer adjacent tissue	-	N.A	2.5+
Indian Patient 56	-	M	66	Squamous cell carcinoma	III	N.A	-
Indian Patient 57	-	M	58	Cancer adjacent tissue	-	N.A	2.5+
Indian Patient 57	-	M	58	Squamous cell carcinoma	III	N.A	-
Indian Patient 58	-	M	51	Cancer adjacent tissue	-	N.A	2+
Indian Patient 58	-	M	51	Squamous cell carcinoma	III	N.A	-
Indian Patient 59	-	M	60	Cancer adjacent tissue	-	N.A	1+
Indian Patient 59	-	M	60	Squamous cell carcinoma	III	N.A	-
Indian Patient 60	-	M	60	Cancer adjacent tissue	-	N.A	2+
Indian Patient 60	-	M	60	Squamous cell carcinoma	III	N.A	-
Indian Patient 61	-	F	68	Cancer adjacent tissue	-	N.A	1.5
Indian Patient 61	-	F	68	Squamous cell carcinoma	II	N.A	-

Pawar et. al., 2013. Downregulation of calcium binding protein S100A9 in Esophageal Squamous Cell Carcinoma. Supplementary Table 1. Summary of IHC labeling for S100A9.

Catalog	Position	Sex	Age	Pathology	Grade	TNM	Score
Indian Patient 62	-	M	42	Cancer adjacent tissue	-	N.A	2+
Indian Patient 62	-	M	42	Squamous cell carcinoma	II	N.A	-
Indian Patient 63	-	M	59	Cancer adjacent tissue	-	N.A	1+
Indian Patient 63	-	M	59	Squamous cell carcinoma	III	N.A	-
Indian Patient 64	-	M	61	Cancer adjacent tissue	-	N.A	1+
Indian Patient 64	-	M	61	Squamous cell carcinoma	III	N.A	-
Indian Patient 65	-	M	64	Cancer adjacent tissue	-	N.A	1+
Indian Patient 65	-	M	64	Squamous cell carcinoma	III	N.A	Few well differentiated keratinized tumor cell 1.5+
Indian Patient 66	-	F	60	Cancer adjacent tissue	-	N.A	1.5+
Indian Patient 66	-	F	60	Squamous cell carcinoma	III	N.A	-
Indian Patient 67	-	M	36	Cancer adjacent tissue	-	N.A	2+
Indian Patient 67	-	M	36	Squamous cell carcinoma	II	N.A	-
Indian Patient 68	-	F	65	Cancer adjacent tissue	-	N.A	2+
Indian Patient 68	-	F	65	Squamous cell carcinoma	II	N.A	-
Indian Patient 69	-	F	60	Cancer adjacent tissue	-	N.A	1+
Indian Patient 69	-	M	60	Squamous cell carcinoma	III	N.A	-
Indian Patient 70	-	M	47	Cancer adjacent tissue	-	N.A	2.5+
Indian Patient 70	-	F	47	Squamous cell carcinoma	III	N.A	-
Indian Patient 71	-	F	50	Cancer adjacent tissue	-	N.A	1+
Indian Patient 71	-	F	50	Squamous cell carcinoma	III	N.A	-
Indian Patient 72	-	M	60	Cancer adjacent tissue	-	N.A	2+
Indian Patient 72	-	M	60	Squamous cell carcinoma	III	N.A	-
Indian Patient 73	-	M	56	Cancer adjacent tissue	-	N.A	2+
Indian Patient 73	-	M	56	Squamous cell carcinoma	II	N.A	Few well differentiated keratinized tumor cell 1.5+
Indian Patient 74	-	M	59	Cancer adjacent tissue	-	N.A	1+
Indian Patient 74	-	M	59	Squamous cell carcinoma	II	N.A	-
Indian Patient 75	-	M	50	Cancer adjacent tissue	-	N.A	2+
Indian Patient 75	-	M	50	Squamous cell carcinoma	III	N.A	Few well differentiated keratinized tumor cell 1+
Indian Patient 76	-	F	38	Cancer adjacent tissue	-	N.A	2+

Pawar et. al., 2013. Downregulation of calcium binding protein S100A9 in Esophageal Squamous Cell Carcinoma. Supplementary Table 1. Summary of IHC labeling for S100A9.

Catalog	Position	Sex	Age	Pathology	Grade	TNM	Score
Indian Patient 76	-	F	38	Squamous cell carcinoma	III	N.A	-
Indian Patient 77	-	M	45	Cancer adjacent tissue	-	N.A	2+
Indian Patient 77	-	M	45	Squamous cell carcinoma	III	N.A	-
Indian Patient 78	-	M	58	Cancer adjacent tissue	-	N.A	1+
Indian Patient 78	-	M	58	Squamous cell carcinoma	III	N.A	-
Indian Patient 79	-	M	55	Cancer adjacent tissue	-	N.A	1+
Indian Patient 79	-	M	55	Squamous cell carcinoma	III	N.A	-
Indian Patient 80	-	F	50	Cancer adjacent tissue	-	N.A	1.5+
Indian Patient 80	-	F	50	Squamous cell carcinoma	II	N.A	Few well differentiated keratinized tumor cell 1.5+
Indian Patient 81	-	F	52	Cancer adjacent tissue	-	N.A	3+
Indian Patient 81	-	F	52	Squamous cell carcinoma	III	N.A	-
Indian Patient 82	-	M	56	Cancer adjacent tissue	-	N.A	3+
Indian Patient 82	-	M	56	Squamous cell carcinoma	III	N.A	-
Indian Patient 83	-	M	55	Cancer adjacent tissue	-	N.A	2+
Indian Patient 83	-	M	55	Squamous cell carcinoma	III	N.A	Few well differentiated keratinized tumor cell 1+
Indian Patient 84	-	M	60	Cancer adjacent tissue	-	N.A	1.5+
Indian Patient 84	-	M	60	Squamous cell carcinoma	III	N.A	-
Indian Patient 85	-	M	50	Cancer adjacent tissue	-	N.A	2+
Indian Patient 85	-	M	50	Squamous cell carcinoma	III	N.A	-
Indian Patient 86	-	F	55	Cancer adjacent tissue	-	N.A	2+
Indian Patient 86	-	F	55	Squamous cell carcinoma	III	N.A	Few well differentiated keratinized tumor cell 1.5+
Indian Patient 87	-	M	50	Cancer adjacent tissue	-	N.A	2+
Indian Patient 87	-	M	50	Squamous cell carcinoma	I	N.A	Few well differentiated keratinized tumor cell 2+
Indian Patient 88	-	M	48	Cancer adjacent tissue	-	N.A	2+
Indian Patient 88	-	M	48	Squamous cell carcinoma	II	N.A	-
Indian Patient 89	-	F	76	Cancer adjacent tissue	-	N.A	2+
Indian Patient 89	-	F	76	Squamous cell carcinoma	III	N.A	-
Indian Patient 90	-	F	70	Cancer adjacent tissue	-	N.A	2+

Pawar et. al., 2013. Downregulation of calcium binding protein S100A9 in Esophageal Squamous Cell Carcinoma. Supplementary Table 1. Summary of IHC labeling for S100A9.

Catalog	Position	Sex	Age	Pathology	Grade	TNM	Score
Indian Patient 90	-	F	70	Squamous cell carcinoma	II	N.A	Few well differentiated keratinized tumor cell 1.5+
Indian Patient 91	-	M	37	Cancer adjacent tissue	-	N.A	2+
Indian Patient 91	-	M	37	Squamous cell carcinoma	II	N.A	-
Indian Patient 92	-	M	50	Cancer adjacent tissue	-	N.A	2+
Indian Patient 92	-	M	50	Squamous cell carcinoma	II	N.A	Few well differentiated keratinized tumor cell 1.5+
Indian Patient 93	-	M	60	Cancer adjacent tissue	-	N.A	2+
Indian Patient 93	-	M	60	Squamous cell carcinoma	I	N.A	Few well differentiated keratinized tumor cell 1+
Indian Patient 94	-	M	70	Cancer adjacent tissue	-	N.A	1.5+
Indian Patient 94	-	M	70	Squamous cell carcinoma	III	N.A	-
Indian Patient 95	-	M	72	Cancer adjacent tissue	-	N.A	2+
Indian Patient 95	-	M	72	Squamous cell carcinoma	III	N.A	-
Indian Patient 96	-	M	40	Cancer adjacent tissue	-	N.A	2+
Indian Patient 96	-	M	40	Squamous cell carcinoma	III	N.A	-
Indian Patient 97	-	M	43	Cancer adjacent tissue	-	N.A	2+
Indian Patient 97	-	M	43	Squamous cell carcinoma	III	N.A	-
Indian Patient 98	-	M	55	Cancer adjacent tissue	-	N.A	1+
Indian Patient 98	-	M	55	Squamous cell carcinoma	III	N.A	-
Indian Patient 99	-	F	50	Cancer adjacent tissue	-	N.A	2+
Indian Patient 99	-	F	50	Squamous cell carcinoma	III	N.A	-
Indian Patient 100	-	M	55	Cancer adjacent tissue	-	N.A	2+
Indian Patient 100	-	M	55	Squamous cell carcinoma	II	N.A	-

# P.S. We have excluded tumor and normal cores that are not paired or in duplicates or have tumor grading information missing from our analysis. The cores for which IHC scoring was excluded in our analysis are indicated in bold font.
